# Circular RNA circNRIP1 promotes migration and invasion in cervical cancer by sponging miR-629-3p and regulating the PTP4A1/ERK1/2 pathway

**DOI:** 10.1038/s41419-020-2607-9

**Published:** 2020-05-26

**Authors:** Xinhui Li, Ningye Ma, Yao Zhang, Heng Wei, Huijie Zhang, Xiaoao Pang, Xiang Li, Dan Wu, Dian Wang, Zhuo Yang, Shulan Zhang

**Affiliations:** Department of Obstetrics and Gynecology, Shengjing Hospital of China Medical University, No. 36, Sanhao Street, Shenyang, Liaoning Province China

**Keywords:** Cervical cancer, Cell invasion, Non-coding RNAs

## Abstract

Emerging evidence indicates that circRNAs play essential roles in tumorigenesis and development. However, the role of circRNAs in cervical cancer (CC) remains unclear. CircRNA microarrays performed on the immortal cervical cell line H8 and the cervical cancer cell line SiHa were used to identify a circRNA, termed circNRIP1 (hsa_circ_0004771), which was upregulated in SiHa. QRT-PCR confirmed that circNRIP1 was upregulated in CC tissues, where its expression was correlated with lymphovascular space invasion. Besides, both in vitro and in vivo experiments demonstrated that circNRIP1 promotes cell proliferation, migration, and invasion. Additionally, we found that miR-629-3p induced tumor suppression by regulating PTP4A1 and the ERK1/2 pathway. Finally, we confirmed that circNRIP1 exerts its effect, at least partially, by sponging miR-629-3p and thereby regulating the PTP4A1/ERK1/2 pathway. Therefore, circNRIP1 may be useful as a potential prognostic biomarker and therapeutic target in CC patients.

## Introduction

According to the National Central Cancer Registry of China (NCCRC), cervical cancer is the sixth most common cancer in women in China, with ~111,000 newly diagnosed cases and nearly 34,000 new deaths in 2015^1^. According to the Surveillance, Epidemiology, and End Results (SEER) registry, between 2009 and 2015, 36% and 15% of women, respectively, were diagnosed at regional and distant stages of cancer progression, despite the availability of viable prevention strategies and vaccines^2^. These women were considered incurable, as they had already lost the opportunity to undergo surgical resection of their lesions due to metastasis^3^. Therefore, it is of utmost importance to clarify the molecular mechanisms underlying the development and progression of CC and to find new, effective therapeutic targets.

Circular RNA (circRNA), a novel class of endogenous noncoding RNA molecules, is characterized by covalently closed loop structures without 5′ to 3′ polarity or a poly A tail^4^. They are abundant, conserved, stable, and display spatio-temporal specificity in different tissues^5–7^. High-throughput sequencing of cervical cancer tissues and cells has shown that numerous circRNAs are differentially expressed in cervical cancer^8–10^. “MiRNA sponging”, in which circRNAs act as ceRNAs to sequester miRNAs away from their target genes, is the most commonly reported role of circRNAs^11^. In cervical cancer, some circRNAs, such as circ_0067934 and circRNA-000284, have been reported to function in the sponging of miRNAs^8,12^.

PTP4A1 (also known as phosphatase of regenerating liver 1), which is reportedly upregulated in many tumor cell lines, including HeLa cells, promotes cell migration and invasion^13–15^. It has been reported that PTP4A1 promotes the activation of Extracellular signal-regulating kinase (ERK) and thereby the expression of Matrix metalloproteinase 2 (MMP2) and MMP9, which, in turn, induces cell migration and invasion^16,17^. ERK signaling, which is a crucial regulator of cell motility, participates in cervical cancer progression^18,19^. MMPs are important hydrolytic enzymes, of which MMP2 and -9 are two of the most widely studied. These are capable of degrading the extracellular matrix, which promotes tumor cell migration, invasion, and distant metastasis^20–22^.

Our study revealed that circNRIP1 promoted cervical cancer cell proliferation, migration, and invasion both in vitro and in vivo, and that miR-629-3p inhibits CC cell progression. Finally, the study indicated that circNRIP1 promotes migration and invasion in cervical cancer by sponging miR-629-3p and regulating the PTP4A1/ERK1/2 pathway.

## Materials and methods

### Ethics approval and consent to participate

Human cervical cancer tissues and adjacent normal tissues were obtained from patients who underwent surgery at Shengjing Hospital of China Medical University. Informed consent was obtained from all patients. BALB/c nude female mice were purchased and housed under special pathogen-free (SPF) conditions. All experiments were approved by the ethics committee of Shengjing Hospital of China Medical University.

### Clinical specimens and cell cultures

Forty cervical cancer tissue and paired adjacent tissue samples were obtained from patients who underwent surgery at Shengjing Hospital of China Medical University from 2018 to 2019. None of the patients had received radiotherapy or chemotherapy prior to surgery. All specimens were stored in liquid nitrogen immediately after resection and subsequently stored at −80 °C until required. Diagnoses were pathologically confirmed by two independent pathologists. Stages were determined based on the updated 2009 FIGO^23^. Cervical cancer cells (HeLa, SiHa, CaSki, and C-33A) were obtained from the Cell Bank of the Chinese Academy of Sciences (Shanghai, China). HeLa cells were cultured in DMEM (High Glucose), CaSki in RPMI 1640 and SiHa and C33A in MEM (Corning, NY, USA) with 10% fetal bovine serum (FBS) and 1% streptomycin and penicillin (GE Healthcare, Chicago, IL, USA). Authentication of cells was verified by short tandem repeat DNA profiling.

### CircRNA microarrays

Arraystar Human circRNA Array v2 analysis was performed by Kangcheng Biotech (Shanghai, China). Sample preparation and microarray hybridization were performed as outlined in the standard protocols stipulated by Arraystar, as described previously^24^. Quantile normalization and subsequent data processing were performed using the R software package. Differentially expressed circRNAs with statistical significance between two groups were identified via Volcano Plot filtering. Differentially expressed circRNAs between two samples were identified through Fold Change filtering. Data associated with circRNA microarray profiling have been deposited in NCBI’s Gene Expression Omnibus^25^ and are accessible through GEO Series accession number GSE147483.

### RNA extraction and qRT-PCR

Total RNA from cells and tissues was isolated using RNAiso Plus (Takara, Dalian, China) according to the manufacturer’s protocol. In order to quantify the amount of mRNA and circRNA, cDNA was synthesized using a PrimeScript™ RT reagent Kit with gDNA Eraser, and qRT-PCR was performed using TB Green™ Premix Ex Taq II (Takara, Dalian, China). The levels of microRNAs were determined using a miRNA 1st Strand cDNA Synthesis Kit and miRNA Universal SYBR qPCR Master Mix (Vazyme biotech, Nanjing, China). Primers were designed and synthesized by Sangon Biotech (Shanghai, China). All primer sequences are listed (Table S1). GAPDH was measured as an endogenous control for mRNA and circRNA, and U6 was used as a control for miRNA. Relative levels of gene expression were calculated using the 2^-ΔΔCt^ method.

### Cellular fraction and RNA isolation

Norgen’s Cytoplasmic & Nuclear RNA Purification Kit (Norgen Biotek Corp, ON, Canada) was used to separately isolate nuclear and cytoplasmic RNA from cultured cells, according to the manufacturer’s protocol.

### Lentivirus vector constructs and cell infection

For in vitro studies, the circNRIP1 overexpression lentivirus vector (OV-cNRIP1) was constructed using the pHBLV-CMV-Circ-MCS-EF1-ZsGreen-T2A-PURO vector purchased from Hanbio Biotechnology (Shanghai, China), and further confirmation was obtained by Sanger Sequencing. We also constructed an empty control vector (OV-NC). Lentiviral vectors containing shRNAs that all covered the backsplicing site of circNRIP1 were also constructed using the pHBLV-U6-MCS-CMV-ZsGreen-PGK-PURO vector, named as sh-cNRIP1. We also constructed a negative shRNA containing only a nonsense sequence, named as sh-NC. The sequences of the shRNAs used are listed (Table S2). Cells were infected with the lentiviral particles, followed by selection via puromycin (8 µg/ml) to select stably-transfected cells.

### Oligonucleotide transfection

Hsa-miR-629-3p mimics, inhibitors and their respective control oligonucleotides were synthesized by Gene-Pharma (Shanghai, China). The sequences of the oligonucleotides used are listed (Table S3). HeLa and SiHa cells were transfected with 50 nM of mimics and 100 nM of inhibitors using Lipofectamine 3000 reagent (Life Technologies, CA, USA) according to the manufacturer’s protocol.

### Wound-healing assay

Cells were cultured in 6-well plates and scraped with the fine end of a 200 µl pipette tip (time 0 h) after they reached 90–100% confluency. Next these were cultured in medium without FBS. Cell migration progress was photographed at 0 h and 48 h following injury. Images of 5 randomly selected fields were obtained using an inverted microscope (TS100-F, Nikon, Japan) with 10× objective lenses. Remodeling was measured as the diminishing area across the induced injury, normalized to the 0 h control, and expressed as migration rate. Experiments were performed in triplicate.

### CCK-8 assay

Cell proliferation was detected using Cell count kit-8 (CCK-8, Dojindo, Japan). Briefly, cells were seeded into 96-well plates at approximately 2000-3000 cells per well, where each group was replicated in quintuplicate. At indicated time points, 10 μl of CCK-8 solution was added to each well. The cells were incubated at 37 °C for another 2 h, following which absorbance at 450 nm was measured using an automatic spectrophotometer (Synergy H1, Bio-Tek, USA). Experiments were performed in triplicate.

### EdU incorporation assay

An EdU incorporation assay was performed using a Cell-Light EdU DNA Cell Proliferation Kit (RiboBio, Guangzhou, China) to detect DNA synthesis and cell proliferation. Cells were seeded in 96-well plates (2 × 10^4^ cells per well). After 36 h, 50 μM of EdU was added to each well and incubated for another 3 h. The cells were then fixed with 4% paraformaldehyde and stained with Apollo Dye Solution. Hoechst 33342 was used to stain the nucleic acid within cells. Images were obtained via inverted fluorescence microscopy with 20× objective lenses, and the numbers of EdU-positive (red) and Hoechst 33342 positive (blue) cells were counted in 5 randomly selected fields. The cell proliferation rate was estimated as being equal to: EdU-positive cells/Hoechst 33342 positive cells. Experiments were performed in triplicate.

### Transwell Matrigel invasion assays

A Matrigel invasion assay was performed using transwell cell culture inserts (8 mm pores; Corning, NY, USA) in 24-well plates. HeLa and SiHa cells (10 × 10^4^) were resuspended in 200 μl of serum-free medium and seeded into the upper chamber of the 24-well plates with Matrigel Matrix (Corning, NY, USA) and incubated for 24 h. Next, 600 μl of medium with 10% FBS was added to the lower chamber as a chemoattractant. Following incubation, the cells were fixed with 4% paraformaldehyde for 30 min and stained with 0.1% crystal violet for 30 min. The number of invading cells was quantified by counting the cells in 5 randomly selected fields using an inverted microscope with 20× objective lenses. Experiments were performed in triplicate.

### Dual-luciferase reporter assay

In order to create luciferase reporter vectors, the sequences of circNRIP1 and PTP4A1–3’UTR and their corresponding mutant versions without miR-629-3p binding sites were synthesized and subcloned into pmirGLO vector (Promega, WI, USA), termed WT-circNRIP1, MUT-circNRIP1, WT-PTP4A1, MUT-PTP4A1, respectively. HEK-293T cells were seeded in 12-well plates at a density of 5 × 10^5^ cells per well for 24 h before transfection. Next, a mixture of miR-629-3p mimics and luciferase reporter vectors was transfected into the HEK-293T cells; each group was treated in triplicate. After 24 h, firefly and Renilla luciferase activity was detected using the Dual-Luciferase Reporter Assay System kit (Promega, WI, USA). Experiments were performed in triplicate.

### Western blot

Cells were lysed in RIPA lysis buffer (Beyotime, Shanghai, China). Equal amounts of protein were separated in 10% SDS-PAGE gels and transferred to PVDF membranes (Millipore, Darmstadt, Germany). The membranes were blocked for 2 h with 5% non-fat dry milk and incubated with primary antibodies overnight at 4 °C. Next, the membranes were incubated with secondary antibodies for 2 h. GAPDH was used as the internal protein loading control. Finally, immune complexes were detected via enhanced chemiluminescence (Beyotime, Shanghai, China) using a GE detection system. Band intensity was quantified by ImageJ software. Each group was treated in triplicate.

### Xenograft model

BALB/c nude female mice (4–5 weeks old) were randomly divided into OV-cNRIP1 and OV-NC groups (6 per group) and housed under standard conditions. Stably-transfected HeLa cells were subcutaneously injected into the right flanks of the mice using 5 × 10^6^ cells per mouse. Tumor volumes were measured every 4–5 d. Three weeks after injection, the mice were humanely euthanized. Subcutaneous tumor tissues were isolated, and the volume and weight of the dissected tumors were measured. Tumor volume was calculated as follows: V (mm^3^) = (length × width^2^)/2. All animal experiments were approved by the ethics committee of Shengjing Hospital of China Medical University.

### Immunohistochemistry (IHC)

Tumor tissue samples (*n* = 3 per group) were fixed with 4% paraformaldehyde and embedded in paraffin. Paraffin sections were incubated overnight with primary antibodies against PTP4A1 (1:50) at 4 °C, followed by incubation with a secondary antibody at room temperature for 1 h and HRP-labeled streptavidin (KIT-9710, MXB Biotechnologies, China) for 10 min. Lastly, the tissues were stained with diaminoaniline (DAB) (ZLI-9018, ZSGB-BIO, China). Five randomly selected fields of sections were photographed using a light microscope (Nikon Eclipse NI, Japan).

### Reagents and antibodies

Anti-TP4A1 (YN2105), anti-ERK1/2 (YT1625), anti-ERK1/2 (Phospho T202/Y204) (YP1197), and anti-MMP9 (YM0446) were purchased from Immunoway (Plano, TX, USA). Anti-GAPDH (60004-1-Ig), anti-MMP2 (66366-1-Ig), anti-E-cadherin (60335-1-Ig), and anti-ZEB2 (14026-1-AP) were purchased from Proteintech (Wuhan, China).

### Statistical analysis

All data were statistically analyzed using GraphPad Prism 8. The results are presented as mean ± standard deviation (SD). Student’s *t*-test (two-tailed), or ANOVA, were performed to analyze 2, or more than 2 groups, respectively. The association between circNRIP1 expression and clinicopathological features was analyzed using a *χ*2 test. The correlation between two variables was determined using the Pearson correlation test. Significant *p* values are represented as follows: *p < 0.05, **p < 0.01, ***p < 0.001, and n.s., no significant difference.

## Results

### CircNRIP1 is upregulated in cervical cancer

**Fig. 1:**
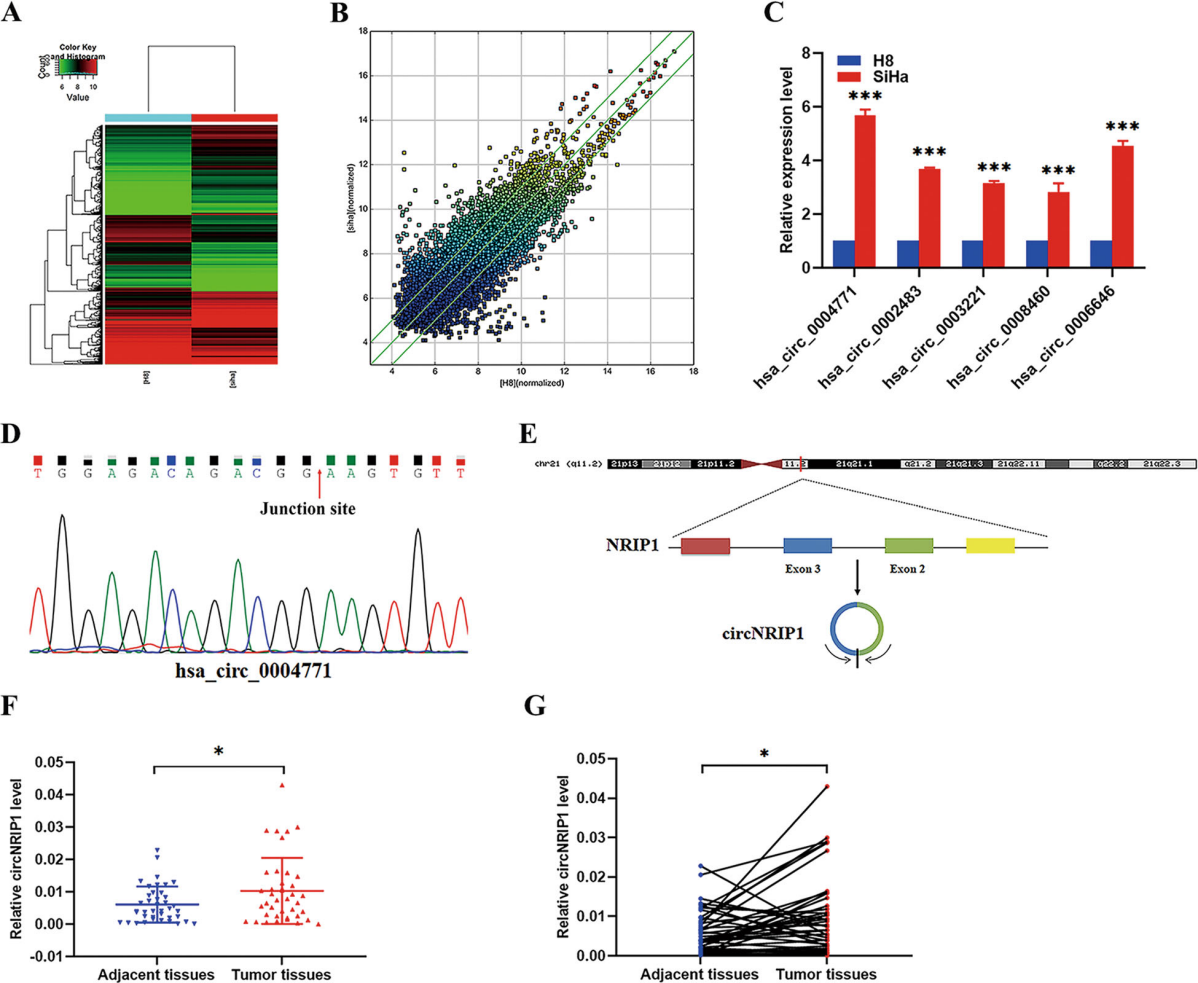
Screening and expression of circNRIP1 in CC cells and tissues. **a** Heat map of all differentially expressed circRNAs between SiHa and H8 cells. Red and green strips represent high and low expression, respectively. **b** The scatter plot was used to assess the differential expression of circRNAs between SiHa and H8 cells. Values on the X and Y axes are normalized signal values of samples (log2 scaled). The green lines are Fold Change Lines. The circRNAs above or below the green line indicate circRNAs with >2.0-fold change between the two samples. **c** Validation of 5 upregulated circRNAs in SiHa and H8 cells using qRT-PCR (*n* = 3). **d** Existing circNRIP1 was validated by qRT-PCR and Sanger sequencing. The red arrow represents the head-to-tail splicing site. **e** Schematic illustration showing that circNRIP1 is formed by head to tail splicing of *NRIP1* exon 2 and exon 3. **f**, **g** Relative expression of circNRIP1 in paired CC and adjacent tissues was measured by qRT-PCR (*n* = 40). Data are presented as mean ± SD. *p < 0.05, ***p < 0.001.

We performed a microarray analysis of the cervical cancer cell line, SiHa, and the immortalized cervical epithelial cell line, H8 (Fig. 1a). Differentially expressed circRNAs between two samples were identified via Fold Change filtering (Fig. 1b). The expression levels of 5 upregulated circRNAs in SiHa and H8 cells were verified using qRT-PCR (Fig. 1c; Table S4). Results obtained from qRT-PCR were consistent with microarray data. Next, head-to-tail splicing of qRT-PCR products was confirmed via Sanger sequencing (Figs. 1d and S1A). Hsa_circ_0004771 displayed the greatest increase and was selected for further study. Hsa_circ_0004771, with a length of 203 bp, is formed by head-to-tail splicing of *NRIP1* exon 2 and exon 3, and is referred to as circNRIP1 (Fig. 1e).

Next, we detected the expression of circNRIP1 in 40 pairs of cervical cancer and adjacent tissue samples. Compared to the adjacent tissues, circNRIP1 expression was significantly higher in the cancer tissues (Fig. 1f, g). Correlation analysis showed that circNRIP1 expression was significantly associated with lymphovascular space invasion (LVSI) in cervical cancer patients (Table 1).

**Table 1:** Correlations between expression of circNRIP1/miR-629-3p and clinicopathological characteristics of cervical cancer tissues.

Characteristics	Group	Cases	circNRIP1 expression			miR-629-3p expression		
			High (20)	Low (20)	*p* value	High (20)	Low (20)	*p* value
Age					0.4053			>0.9999
	≥60	7	2	5		4	3	
	<60	33	18	15		16	17	
HPV type					0.1843			0.3690
	16/18	32	18	14		18	14	
	Others	3	0	3		1	2	
	No	5	2	3		1	4	
Histologic grade					0.5421			0.7384
	Well	3	1	2		1	2	
	Moderate	31	17	14		15	16	
	Poor	6	2	4		4	2	
Histologic type					0.6614			0.6614
	Squamous	33	17	16		17	16	
	Adenocarcinoma	5	3	2		3	2	
	Others	2	0	2		0	2	
Tumor size					0.1025			0.7440
	≥4 cm	15	5	10		8	7	
	<4 cm	25	15	10		12	13	
FIGO stage					0.1967			0.0239*
	Stage I	24	14	10		16	8	
	Stage II	16	6	10		4	12	
Lymphatic metastasis					0.6579			0.6579
	Yes	6	2	4		2	4	
	No	34	18	16		18	16	
Stromal invasion					0.3416			0.6326
	≥1/2	35	19	16		18	17	
	<1/2	5	1	4		2	3	
LVSI					0.0114*			0.5271
	Yes	20	14	6		11	9	
	No	20	6	14		9	11	

*p < 0.05.

### CircNRIP1 promotes cervical cancer proliferation, migration, and invasion in vitro

**Fig. 2:**
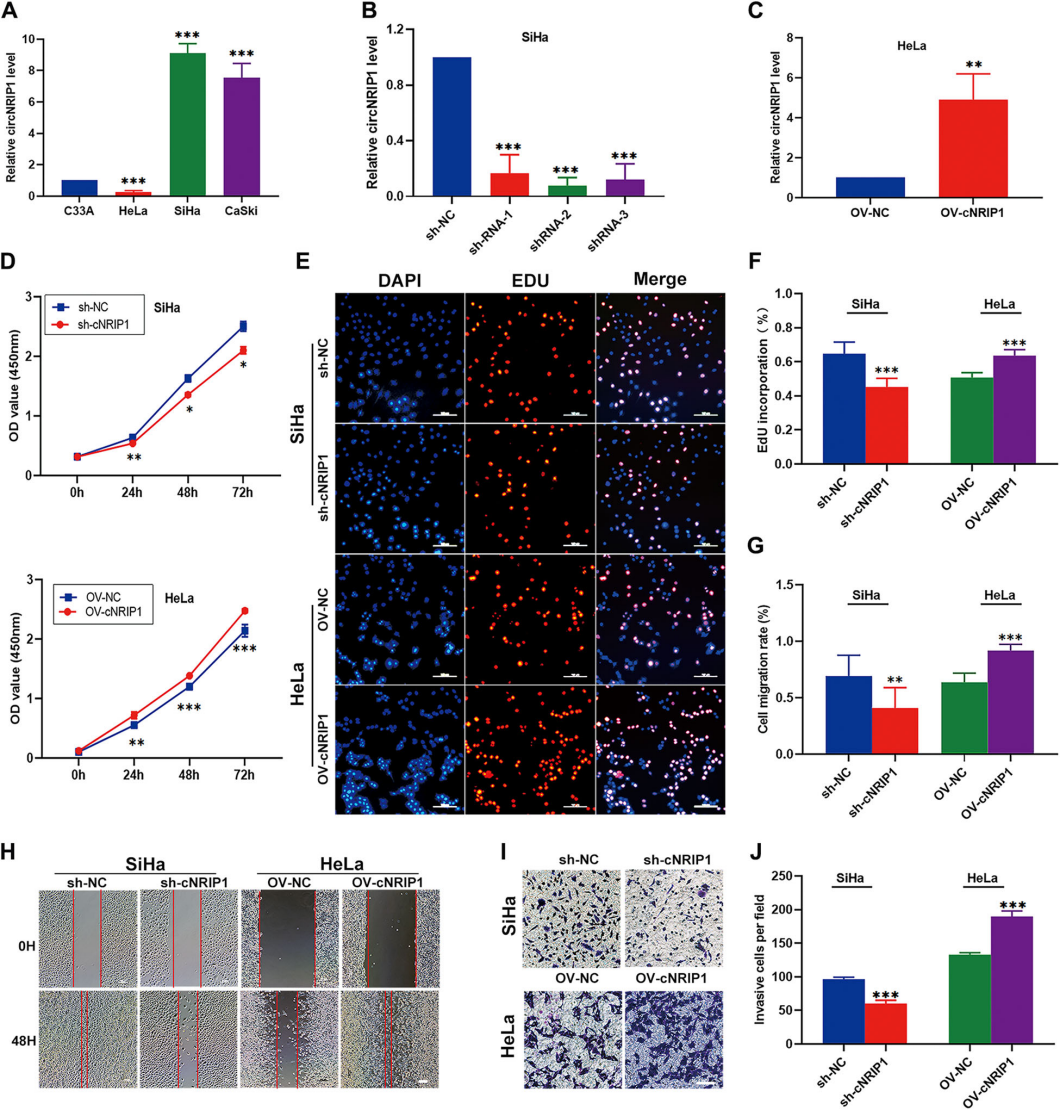
CircNRIP1 promotes CC cell proliferation, migration, and invasion in vitro. **a** Relative expression of circNRIP1 in 4 cervical cancer cell lines was determined by qRT-PCR (*n* = 3). **b**, **c** QRT-PCR was performed to verify the silencing efficiency of three independent shRNAs targeting circNRIP1 in SiHa cells and the overexpression of circNRIP1 in HeLa cells transfected with the OV-circNRIP1 vector (*n* = 3). **d–f** CCK8 and EdU assays were used to analyze proliferation of CC cells following transfection with sh-cNRIP1 or OV-cNRIP1 vectors (*n* = 3). **g**, **h** The effect of circNRIP1 on cell migration was evaluated using a wound-healing assay in stably-transfected sh-cNRIP1 or OV-cNRIP1 CC cells (*n* = 3). **i**, **j** Invasive capabilities of SiHa and HeLa cells transfected with sh-cNRIP1 or OV-cNRIP1 vectors were evaluated via transwell Matrigel invasion assays (*n* = 3). Scale bar, 100 μm; data are presented as mean ± SD. *p < 0.05, **p < 0.01, ***p < 0.001.

To explore the role of circNRIP1 in cervical cancer, we examined circNRIP1 expression in 4 CC cell lines to determine cell lines that could be used for silencing and overexpression studies. The expression of circNRIP1 was highest in SiHa (HPV16+) and lowest in HeLa (HPV18+) (Fig. 2a). Therefore, the HeLa cell line was selected for overexpression of circNRIP1 and the SiHa cell line for silencing. Next, we constructed 3 shRNA vectors to knockdown circNRIP1 expression. The vectors were successfully transfected into SiHa cells (Fig. S1B). Following the transfection shRNAs into cells, silencing efficiency of the shRNAs was evaluated using qRT-PCR, following which shRNA-2 was selected for subsequent experiments due to its high inhibitory efficiency (Fig. 2b). The circNRIP1 overexpression lentiviral vector was also successfully transfected into HeLa cells (Fig. S1B), and its ability to overexpress circNRIP1 was confirmed via qRT-PCR (Fig. 2c).

Next, we examined the effect of circNRIP1 silencing on cervical cancer cells. CCK-8 and EdU assays indicated that SiHa cell proliferation was suppressed by sh-cNRIP1 (Fig. 2d–f). Wound healing and transwell assays showed that downregulation of circNRIP1 significantly decreased cell migration and invasion ability (Fig. 2g–j). Moreover, overexpression of circNRIP1 promoted proliferation, migration, and invasion of HeLa cells compared to the OV-NC group (Fig. 2d–j). These results suggested that circNRIP1 promotes cell proliferation, migration, and invasion in cervical cancer cells.

### CircNRIP1 acts as a sponge for miR-629-3p

**Fig. 3:**
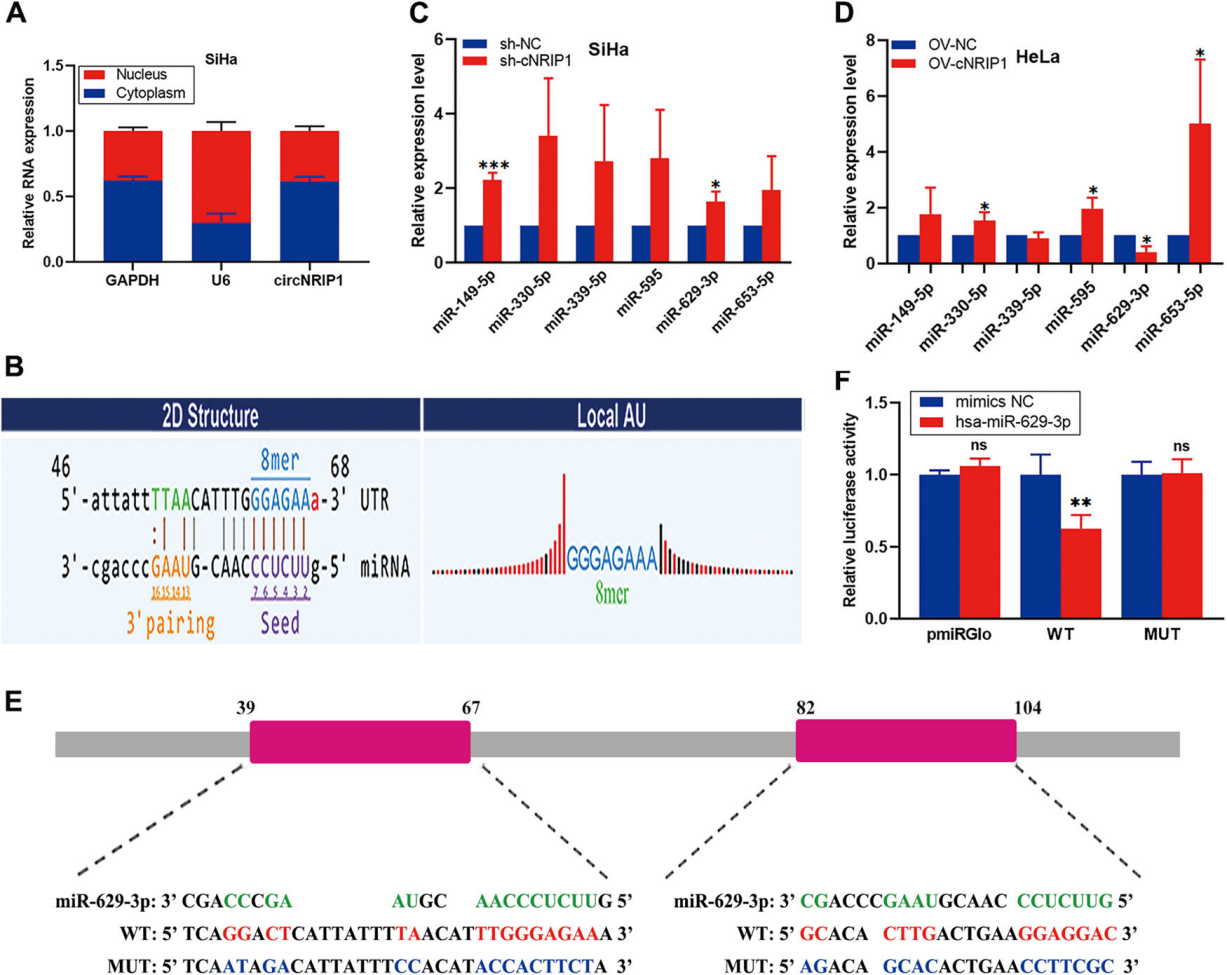
CircNRIP1 acts as a sponge for miR-629-3p. **a** Cytoplasmic and nuclear RNA was isolated and measured using qRT-PCR; circNRIP1 expression was also measured (*n* = 3). **b** Potential binding sites between circNRIP1 and miR-629-3p predicted with Arraystar’s miRNA target prediction software. **c**, **d** QRT-PCR was used to confirm that circNRIP1 negatively regulates miR-629-3p expression (*n* = 3). **e**, **f** A dual-luciferase reporter assay was performed to measure direct binding between circNRIP1 and miR-629-3p based on their complementary sequences (*n* = 3). All data are presented as mean ± SD. *p < 0.05, **p < 0.01, **p < 0.001.

It has been widely reported that circRNAs may function as “miRNA sponges” in cancer cells. To explore the molecular mechanism underlying the functioning of circNRIP1, we isolated cytoplasmic and nuclear RNA from SiHa cells. Through qRT-PCR, we found that circNRIP1 was mostly expressed in cytoplasm (Fig. 3a). Next, using Arraystar’s home-made miRNA target prediction software based on TargetScan^26^ and miRanda^27^, we detected 5 potential circNRIP1-binding microRNAs, namely miR-330-5p, miR-339-5p, miR-595, miR-629-3p and miR-653-5p (Figs. 3b, S1C). Next, we examined the effect of circNRIP1 on the expression levels of these 5 miRNAs. The results showed that the expression of miR-629-3p was decreased in OV-cNRIP1 cells but increased in sh-cNRIP1 cells, compared to their respective negative controls (Fig. 3c, d). This indicated that circNRIP1 may regulate miR-629-3p expression. In order to further confirm this interaction, we conducted a dual-luciferase reporter assay in 293T cells. We used the RNAhybrid bioinformatics prediction tool to calculate the secondary conformation of circNRIP1 and miR-629-3p^28^ (Fig. S2). WT-circNRIP1 and MUT-circNRIP1 sequences were cloned into the pmirGLO vector (Fig. 3e). The results showed that miR-629-3p mimics significantly decreased luciferase activity of the WT groups but not of the MUT groups, suggesting that circNRIP1 may directly interact with miR-629-3p (Fig. 3f). Considered together, these results suggest that circNRIP1 acts as a sponge for miR-629-3p.

### MiR-629-3p inhibits CC cell proliferation, migration, and invasion by inhibiting PTP4A1 in vitro

**Fig. 4:**
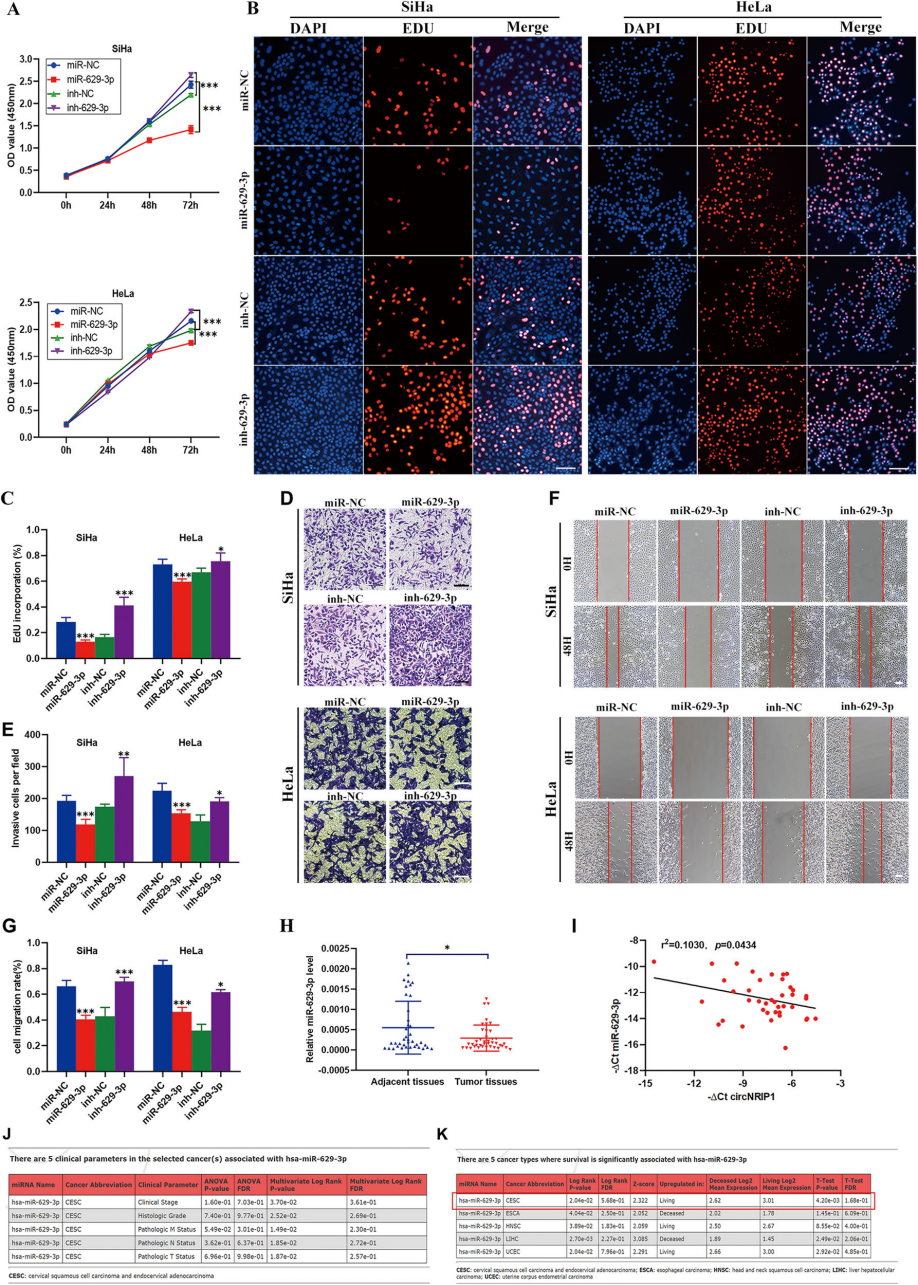
MiR-629-3p inhibits proliferation, migration, and invasion of CC cells. **a–c** CCK8 and EdU assays were used to analyze the proliferation of CC cells following transfection with miR-629-3p and inh-629-3p or respective controls (*n* = 3). **d**, **e** Wound-healing assays were performed to evaluate cell migration capability following transfection with miR-629-3p and inh-629-3p or respective controls (*n* = 3). **f**, **g** Cell invasion ability of CC cells transfected with miR-629-3p and inh-629-3p or respective controls were evaluated using transwell Matrigel invasion assays (*n* = 3). **h** Relative expression of miR-629-3p in paired CC and adjacent tissues was measured by qRT-PCR (*n* = 40). **i** Negative correlation between circNRIP1 expression and miR-629-3p (*n* = 40). **j** Results of analyses from OncomiR showing the association between miR-629-3p and the clinical parameters of CESC. **k** Results of analyses from the OncomiR showing that miR-629-3p is associated with the survival of CESC patients. Scale bar, 100 μm; data are presented as mean ± SD. *p < 0.05, **p < 0.01, **p < 0.001.

To our knowledge, miR-629-3p has not been reported on in the context of cervical cancer. In order to investigate the function of miR-629-3p in cervical cancer cells, miR-629-3p mimics, inhibitors (inh-629-3p), and their respective controls were transfected into cells. The qRT-PCR results indicated that miR-629-3p was successfully upregulated or downregulated following transfection with the mimics or inhibitors, respectively (Fig. S3A, B). Next, cell viability and proliferation were determined via CCK8 assay and EdU assay, and the results indicated that upregulation of miR-629-3p inhibited proliferation of cells while downregulation of miR-629-3p promoted proliferation, compared with the controls (Fig. 4a–c). Wound-healing assay and transwell assay results indicated that, whereas miR-629-3p mimics significantly inhibited the migration and invasion of CC cells, inh-629-3p induced the opposite (Fig. 4d–g). Furthermore, qRT-PCR results suggested that miR-629-3p was downregulated in human cervical cancer tissues compared to adjacent normal tissues (Fig. 4h). Further analysis indicated that miR-629-3p expression was negatively correlated to circNRIP1 expression (Fig. 4i), while its expression was significantly associated with the FIGO stage in cervical cancer patients (Table 1). Next, we used OncomiR, an online pan-cancer resource for analysis of miRNA dysregulation, to analyze the association between miR-629-3p and cervical cancer^29^. The results also showed that miR-629-3p was associated with the clinical stage and histologic grade of CC patients (Fig. 4j). Besides, higher levels of miR-629-3p were correlated with better prognoses in CESC (Cervical squamous cell carcinoma and endocervical adenocarcinoma) (Fig. 4k).

**Fig. 5:**
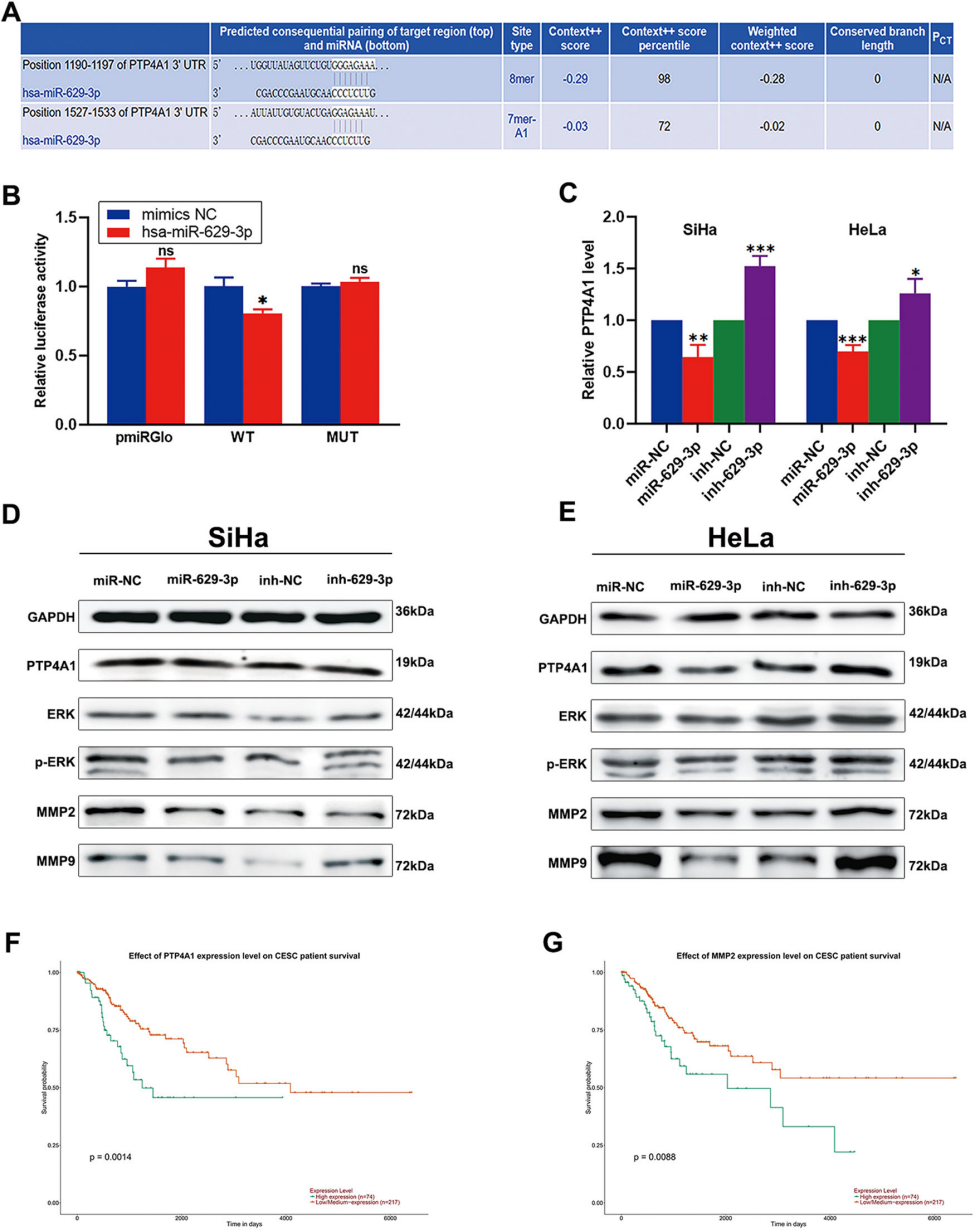
MiR-629-3p acts by targeting PTP4A1 and regulating the ERK1/2 pathway. **a** Potential binding sites between miR-629-3p and PTP4A1 that were predicted via the TargetScan database. **b** A dual-luciferase reporter assay was performed to measure direct binding between miR-629-3p and PTP4A1 based on their complementary sequences (*n* = 3). **c** QRT-PCR was used to confirm that miR-629-3p negatively regulates PTP4A1 expression at the RNA level (*n* = 3). **d**, **e** Western blot was performed to confirm that miR-629-3p acts by targeting PTP4A1 and regulating the ERK1/2 pathway at the protein level (*n* = 3). **f**, **g** Kaplan–Meier survival analysis of PTP4A1 and MMP2 based on TCGA data (*n* = 291). All data are presented as mean ± SD. *p < 0.05, **p < 0.01, **p < 0.001.

Reportedly, miRNAs function by post-transcriptionally regulating target mRNAs via complementary binding. Bioinformatics predictions indicated that miR-629-3p possesses binding sites that are potentially complementary to PTP4A1 (Fig. 5a). We conducted luciferase reporter assays to verify this interaction. The results showed that transfection of miR-629-3p mimics significantly reduced the activity of WT-PTP4A1 but did not affect the activity of MUT-PTP4A1 (Figs. 5b, S4). Furthermore, qRT-PCR and western blot assays showed that miR-629-3p suppressed PTP4A1 expression at both mRNA and protein levels (Figs. 5c–e, S3C). Together, these results suggest that *PTP4A1* is a target of miR-629-3p. Previous studies have reported that PTP4A1 can promote cancer migration and invasion via the ERK1/2 signaling pathway^16,17^. Therefore, we detected the expression of ERK1/2, MMP2, and MMP9 using western blot. Results showed that miR-629-3p overexpression decreased the expression of MMP2 and MMP9 via the ERK1/2 pathway, while inh-629-3p induced the opposite (Figs. 5d, e, S3D–G). Besides, a Kaplan–Meier survival curve, generated using the UALCAN cancer database based on TCGA (The Cancer Genome Atlas), showed that higher levels of PTP4A1 and MMP2 were correlated with poorer prognoses^30^ (Fig. 5f, g). In conclusion, these results revealed that miR-629-3p significantly inhibited the migration and invasion of cervical cancer cells by targeting PTP4A1 via the ERK1/2 pathway.

### CircNRIP1 promotes CC cell progression by sponging miR-629-3p

**Fig. 6:**
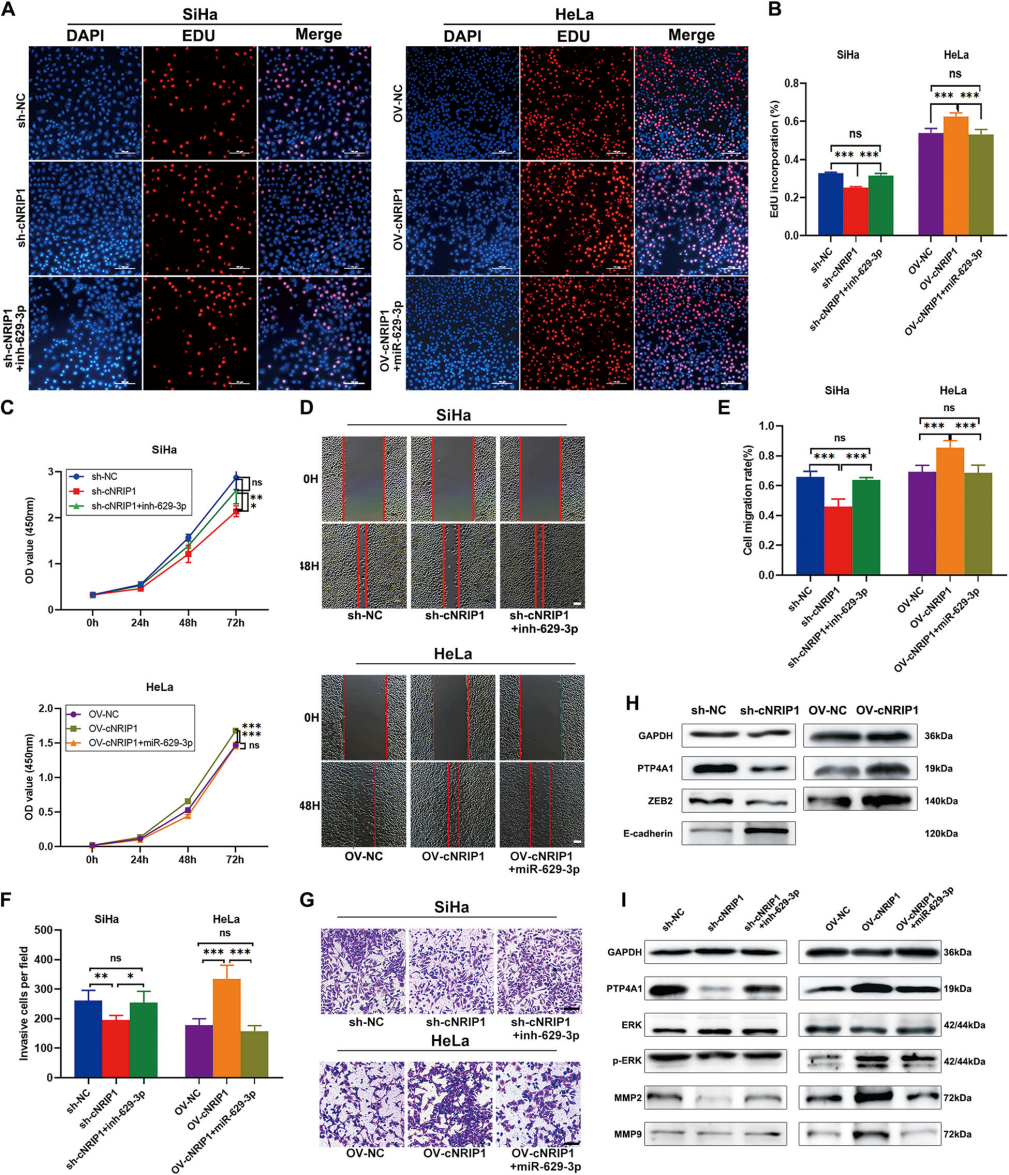
CircNRIP1 promotes CC cell progression by sponging miR‐629-3p. **a–c** EdU assays and CCK8 assays were performed to analyze the proliferation of circNRIP1 overexpression and knockdown cells following transfection with miR-629-3p or inh-629-3p (*n* = 3). **d**, **e** Wound-healing assays were performed to evaluate the effects of circNRIP1 overexpression and knockdown on cell migration following transfection with miR-629-3p or inh-629-3p (*n* = 3). **f**, **g** Invasion capability of circNRIP1-overexpressing and knockdown cells transfected with either miR-629-3p or inh-629-3p were evaluated by transwell Matrigel invasion assays (*n* = 3). **h** CircNRIP1 regulates PTP4A1, E-cadherin and ZEB2 expression (Expression of E-cadherin was not detected in HeLa cells); (*n* = 3). **i** Western blot was used to confirm that miR-629-3p reverses the effects of circNRIP1 on PTP4A1 expression and the ERK1/2 pathway (*n* = 3). Scale bar, 100 μm; data are presented as mean ± SD. *p < 0.05, **p < 0.01, **p < 0.001.

In order to further verify whether circNRIP1 promotes CC cell progression by sponging miR-629-3p, HeLa cells were co-transfected with OV-cNRIP1 and miR-629-3p and SiHa cells were co-transfected with sh-cNRIP1 and inh-629-3p. The results of CCK8 and EdU assays suggested that miR-629-3p mimics inhibited the effect of circNRIP1 on HeLa cells and that inh-629-3p reinstated the proliferative capacity of sh-circNRIP1 cells (Fig. 6a–c). Moreover, wound healing and transwell assays also showed that miR-629-3p could reverse the effects of circNRIP1 overexpression and that inh-629-3p could reinstate the effects of circNRIP1 knockdown (Fig. 6d–g).

Since these findings suggested that circNRIP1 may function in cervical cancer cells by sponging miR-629-3p, we examined whether circNRIP1 regulates the expression of *PTP4A1*, the target gene of miR-629-3p. Western blot results showed that upregulation of circNRIP1 increased PTP4A1 expression, while downregulation of circNRIP1 decreased PTP4A1 expression (Figs. 6h, S3H). We examined whether miR-629-3p would rescue the effects of circNRIP1 on PTP4A1 expression levels. Transfecting HeLa/OV-cNRIP1 with miR-629-3p reversed PTP4A1 upregulation, while inh-629-3p rescued PTP4A1 downregulation in SiHa/sh-cNRIP1 cells (Figs. 6i, S3K). p-ERK1/2, MMP2, and MMP9 expression changes were consistent with those of PTP4A1 (Figs. 6i, S3L–O).

Previous studies have reported that circNRIP1 functions by sponging miR-149-5p or miR-653, and promoting cell epithelial–mesenchymal transition (EMT)^31,32^. Thus, in order to understand the molecular mechanism of circNRIP1 more comprehensively, we examined the expression of miR-149-5p and miR-653 in different groups. Results showed that miR-149-5p was upregulated in sh-cNRIP1 group while miR-653 was also upregulated rather than downregulated in the OV-cNRIP1 group (Fig. 3c, d). Meanwhile, circNRIP1 also increased ZEB2 expression and decreased E-cadherin expression in CC cells, indicating that circNRIP1 was also involved in EMT in cervical cancer (Figs. 6h, S3I–J). Considered together, these findings support the hypothesis that circNRIP1 promotes cervical cancer cell migration and invasion, at least partially, by sponging miR-629-3p and regulating the PTP4A1/ERK1/2 pathway.

### CircNRIP1 promotes the growth of cervical cancer tumors in vivo

**Fig. 7:**
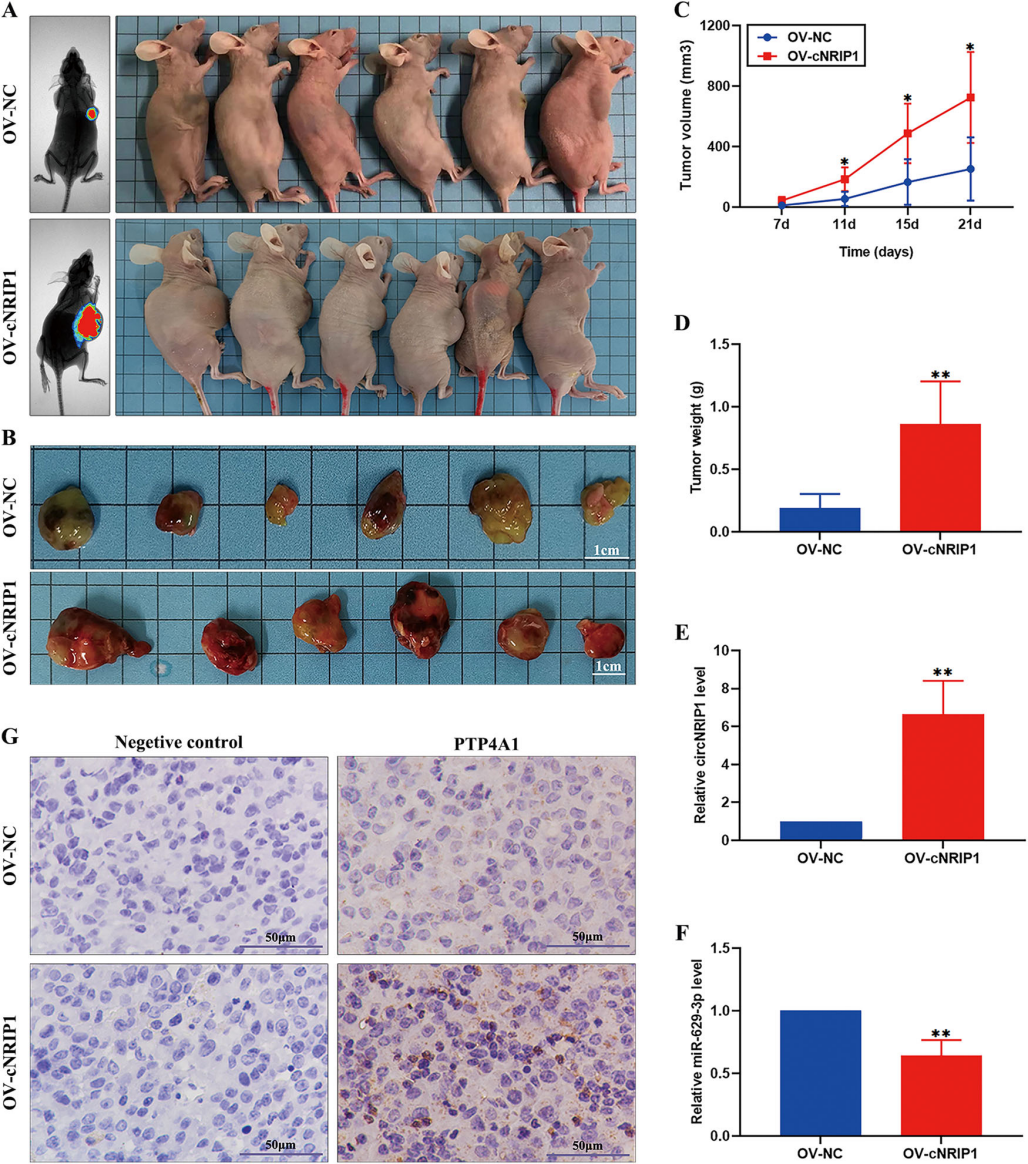
CircNRIP1 promotes the growth of cervical cancer tumors in vivo. **a** HeLa/OV-cNRIP1 and HeLa/OV-NC cells were injected into nude mice and successfully established subcutaneous xenograft tumors. **b** Representation of the xenograft tumor in nude mice (*n* = 6 for each group). Scale bar, 1 cm. **c**, **d** Compared to the control group, tumor size, and weight were significantly increased in the OV-cNRIP1 group (*n* = 6). **e**, **f** CircNRIP1 and miR-629-3p expression levels were measured using qRT-PCR in both groups (*n* = 3). **g** Expression of PTP4A1 was measured by IHC (*n* = 3), scale bar, 50 μm. Data are presented as mean ± SD. *p < 0.05, **p < 0.01, **p < 0.001.

**Fig. 8:**
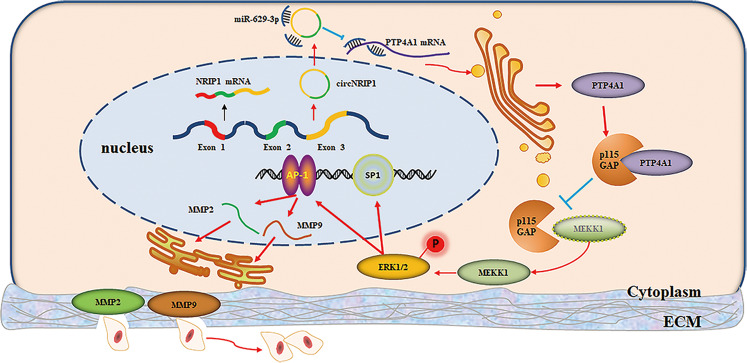
Schematic diagram of the circNRIP1/miR-629-3p/PTP4A1 pathway in CC cells. CircNRIP1 promotes cell migration and invasion in cervical cancer by sponging miR-629-3p and regulating the PTP4A1/ERK1/2 pathway.

To further investigate whether circNRIP1 regulated tumor growth in vivo, HeLa cells transfected with OV-cNRIP1 or OV-NC were injected subcutaneously into BALB/c nude female mice (Fig. 7a). Tumor volumes were monitored 7 d following injection. The mice were humanely euthanized 3 weeks following injection, subcutaneous tumor tissues isolated, and the volume and weight of dissected tumors measured. Overexpression of circNRIP1 significantly promoted tumor growth in vivo (Fig. 7b–d). Furthermore, qRT-PCR (3 samples per group) showed that circNRIP1 was upregulated and miR-629-3p was downregulated in the OV-cNRIP1 group (Fig. 7e, f). IHC (3 samples per group) confirmed that PTP4A1 was elevated in the tumor tissues of the OV-cNRIP1 group, compared with those of the control group (Fig. 7g). These results may indicate that circNRIP1 promoted the progression of cervical cancer by, at least in part, sponging miR-629-3p and regulating the PTP4A1/ERK1/2 pathway (Fig. 8)^16,17^.

## Discussion

In recent years, numerous studies have confirmed that circRNAs, which regulate various biological processes, play several roles in carcinogenesis and cancer progression^33,34^. Furthermore, circRNAs may be of use as potential biomarkers and therapeutic targets for cancers due to their special stable loop structure^35,36^. However, the expression profile and function of circRNAs in cervical cancer remains unclear. The current study performed a microarray analysis on SiHa and H8 cell lines and identified a novel circRNA, termed circNRIP1, which was significantly upregulated in the SiHa cell. Next, we validated the differential expression between H8 and SiHa using qRT-PCR. We further quantified the expression of circNRIP1 in tumor tissues obtained from cervical cancer patients. The results revealed that circNRIP1 was upregulated in tumor tissues and that its expression was significantly correlated with LVSI. LVSI is an important intermediate risk factor used to guide adjuvant treatment decisions and fertility-sparing approaches, which is difficult to assess before surgery^3,37,38^. Thus, circNRIP1 may be a good indicator of LVSI, and thereby guide CC treatment. However, the sample size used in our study was small and did not include samples from patients in advanced stages of cancer who had lost the opportunity to qualify for surgical resection of the lesions due to metastasis. Besides, circNRIP1 expression was detected only in tumor tissues, and not in serum, which may be more suitable for biomarker detection^39^. Additionally, cervical cytology has long been used to test for cervical neoplasia. Many studies have used exfoliated cervical cells to detect cancer-specific gene methylation^40,41^. Therefore, liquid-based cytology samples may be more suitable for detection of circRNAs due to the non-invasiveness associated with this technique. In summary, further clinical studies using larger sample sizes and a wider variety of samples, including tumor tissues, serum, and cervical liquid-based cytology are needed to evaluate the clinical value of circNRIP1 as a biomarker.

Next, gain- and loss-of-function experiments demonstrated that circNRIP1 may promote cervical cancer cell proliferation, migration, and invasion both in vitro and in vivo. Growing evidence has shown that circRNAs may function in the sponging of miRNAs in cervical cancer^42–44^. A dual-luciferase assay and qRT-PCRs, performed by the current study, confirmed that circNRIP1 may sponge miR-629-3p. MiR-629-3p has been reported to promote lung carcinoma and breast cancer progression, but has not been reported on in the context of cervical cancer^45,46^. Interestingly, in our study, gain- and loss-of-function experiments in vitro showed that miR-629-3p inhibited cervical cancer cell proliferation, migration, and invasion. MiR-629-3p expression was downregulated in tumor tissues, and higher levels of miR-629-3p were correlated with better prognoses in CESC. Moreover, we found a new target gene of miR-629-3p termed *PTP4A1*. PTP4A1 reportedly promotes ERK1/2 activation and thereby the expression of MMP2 and -9, which, in turn, induces cell migration and invasion^13–17^. Thus, we hypothesized that miR-629-3p acts as a tumor suppressor in CC by targeting *PTP4A1* and regulating the ERK1/2 pathway. Our analysis of the expression levels of these proteins in different cell groups confirmed this hypothesis.

Based on the above results, we hypothesized that circNRIP1 functions by regulating the miR-629-3p/PTP4A1/ERK1/2 axis. Further rescue experiments showed that miR-629-3p rescued the phenotypes of cells overexpressing circNRIP1, and that inh-629-3p restored the effects of circNRIP1 knockdown on cell function. Moreover, circNRIP1 also enhanced the oncogenic effect of PTP4A1 and consequently activated ERK1/2. Considered together, our findings indicated that circNRIP1 functions by sponging miR-629-3p and regulating its target *PTP4A1* to promote cell migration and invasion. Besides, circRNAs reportedly regulate cell growth by sponging multiple miRNAs^47,48^. Our results also indicated that miR-149-3p may also function in SiHa cells but not in HeLa cells. Therefore, our results could only conclude that circNRIP1 functions, at least partially, by sponging miR-629-3p in cervical cancer. Thus, we inferred that circNRIP1 may act as an oncogene in multiple cancers, functioning by sponging different miRNAs or even sponging multiple miRNAs simultaneously due to the specific cells or tissues involved^31,32^. Many studies have reported that ceRNA-mediated regulation may be determined by the number of transcriptomic miRNA-binding sites and miRNA abundance^49,50^. Therefore, we propose that these results may due to the differences in the abundance of circNRIP1, miRNAs, and miRNA targets in different cell lines. However, the mechanism via which circNRIP1 changes its mode of function in different cancers requires further exploration.

In conclusion, our results indicated that circNRIP1 is significantly upregulated in CC tissues and promotes migration and invasion in CC, at least partially, by sponging miR-629-3p and regulating the PTP4A1/ERK1/2 pathway. Our results provide additional evidence indicating that circRNAs function as miRNA sponges, suggesting that circNRIP1 may be a potential prognostic biomarker and therapeutic target for CC.

## Supplementary information

Supplementary Figure Legends

Figure S1

Figure S2

Figure S3

Figure S4

Table S1-S4
