# Supplementary material for: Circular RNA circNRIP1 promotes migration and invasion in cervical cancer by sponging miR-629-3p and regulating the PTP4A1/ERK1/2 pathway
Source: Cell Death Dis. 2020 May 26;11(5):399. doi: 10.1038/s41419-020-2607-9 (PMC7251091; doi:10.1038/s41419-020-2607-9)
Supplement: Supplementary file 1 — Supplementary Figure Legends [file 41419_2020_2607_MOESM1_ESM.docx]

**Figure S1 (A**) The presence of 4 upregulated circRNAs was validated by Sanger sequencing. (**B**) Lentiviral vectors with GFP were successfully transfected into cells. (**C**) Prediction of potential circNRIP1-binding microRNAs using Arraystar's home-made miRNA target prediction software.

**Figure S2** Result of microRNA target prediction of circNRIP1 based on RNA hybrid.

**Figure S3** **(A, B**) Transfection efficiency of miR-629-3p, inh-629-3p, and the respective controls was confirmed by qRT-PCR (n=3). **(C-G)** Analysis of PTP4A1, ERK1/2, p-ERK1/2, MMP2, MMP9 protein expression in CC cells following miR-629-3p knockdown, or overexpression, using western blot (n=3). **(H-J)** Analysis of PTP4A1, E-cadherin, and ZEB2 protein expression in CC cells following circNRIP1 knockdown, or overexpression, using western blot (Expression of E-cadherin was not detected in HeLa cells); (n=3). **(K-O)** Western blot was used to confirm that miR-629-3p may reverse the effects of circNRIP1 on PTP4A1 expression and the ERK1/2 pathway (n=3).

**Figure S4** Sequences of WT-PTP4A1 and MUT-PTP4A1.
