# Supplementary figures and images for: Circular RNA circNRIP1 promotes migration and invasion in cervical cancer by sponging miR-629-3p and regulating the PTP4A1/ERK1/2 pathway
Source: Cell Death Dis. 2020 May 26;11(5):399. doi: 10.1038/s41419-020-2607-9 (PMC7251091; doi:10.1038/s41419-020-2607-9)

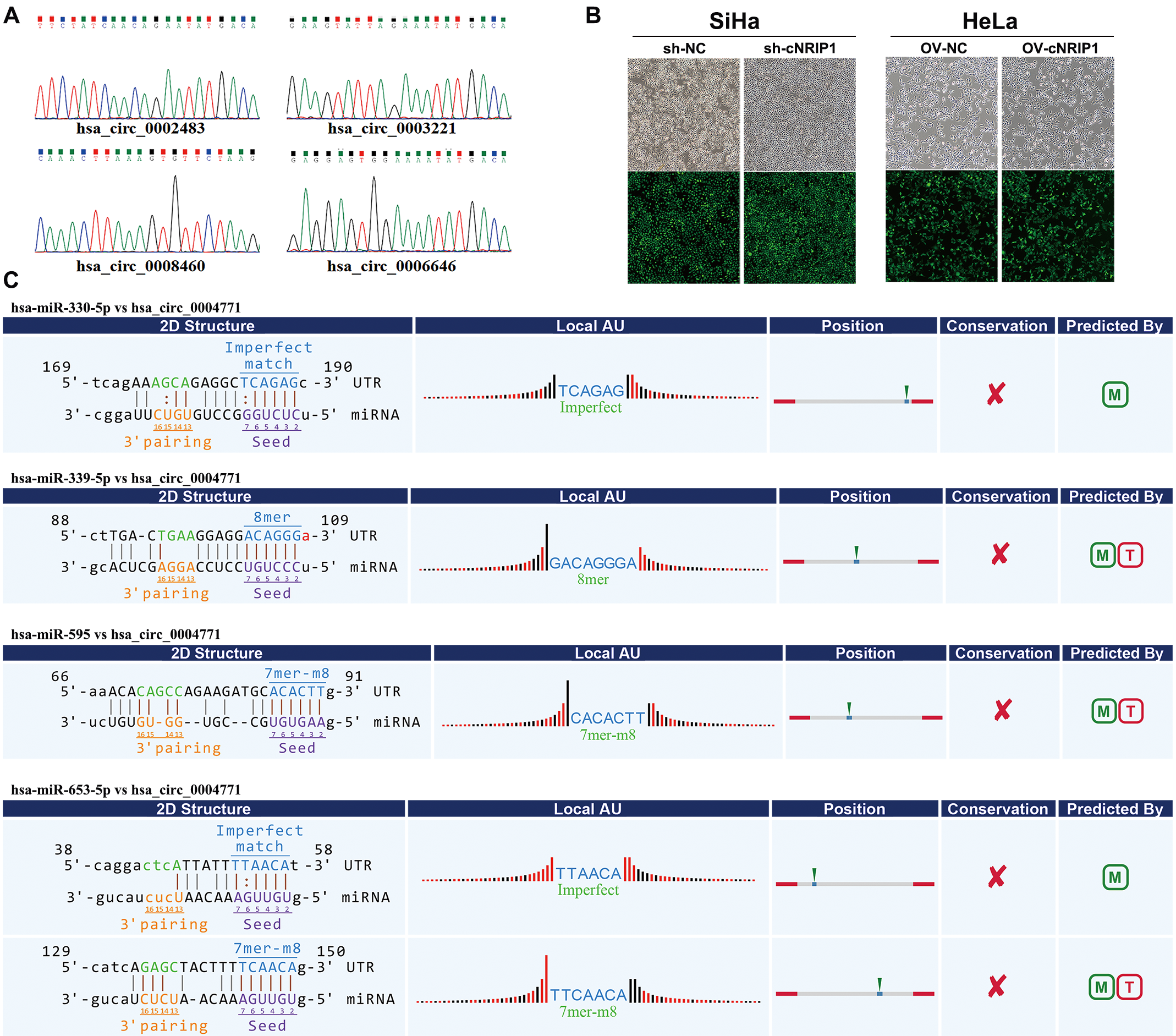

Supplement: Supplementary file 2 — Figure S1 [file 41419_2020_2607_MOESM2_ESM.png]

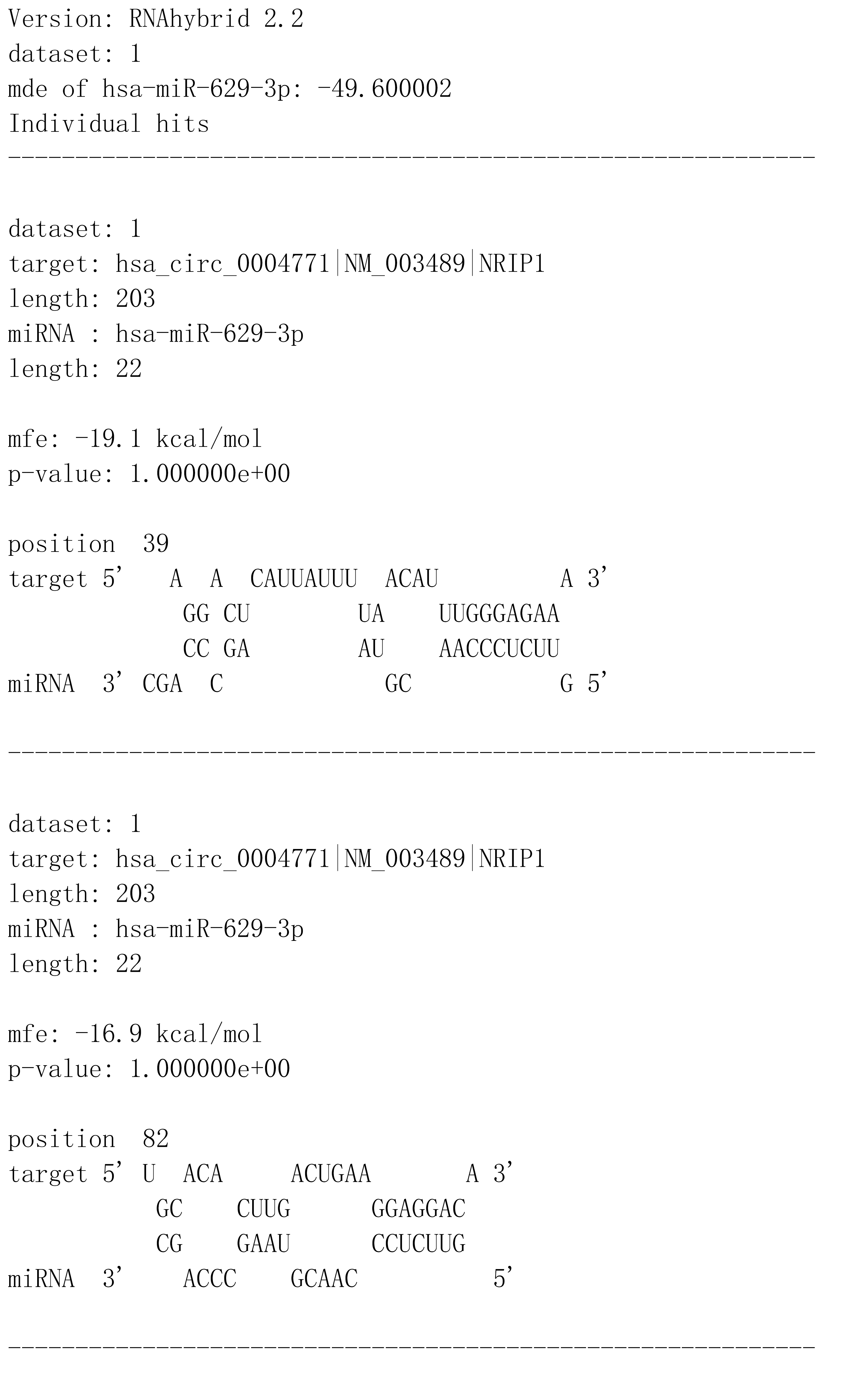

Supplement: Supplementary file 3 — Figure S2 [file 41419_2020_2607_MOESM3_ESM.png]

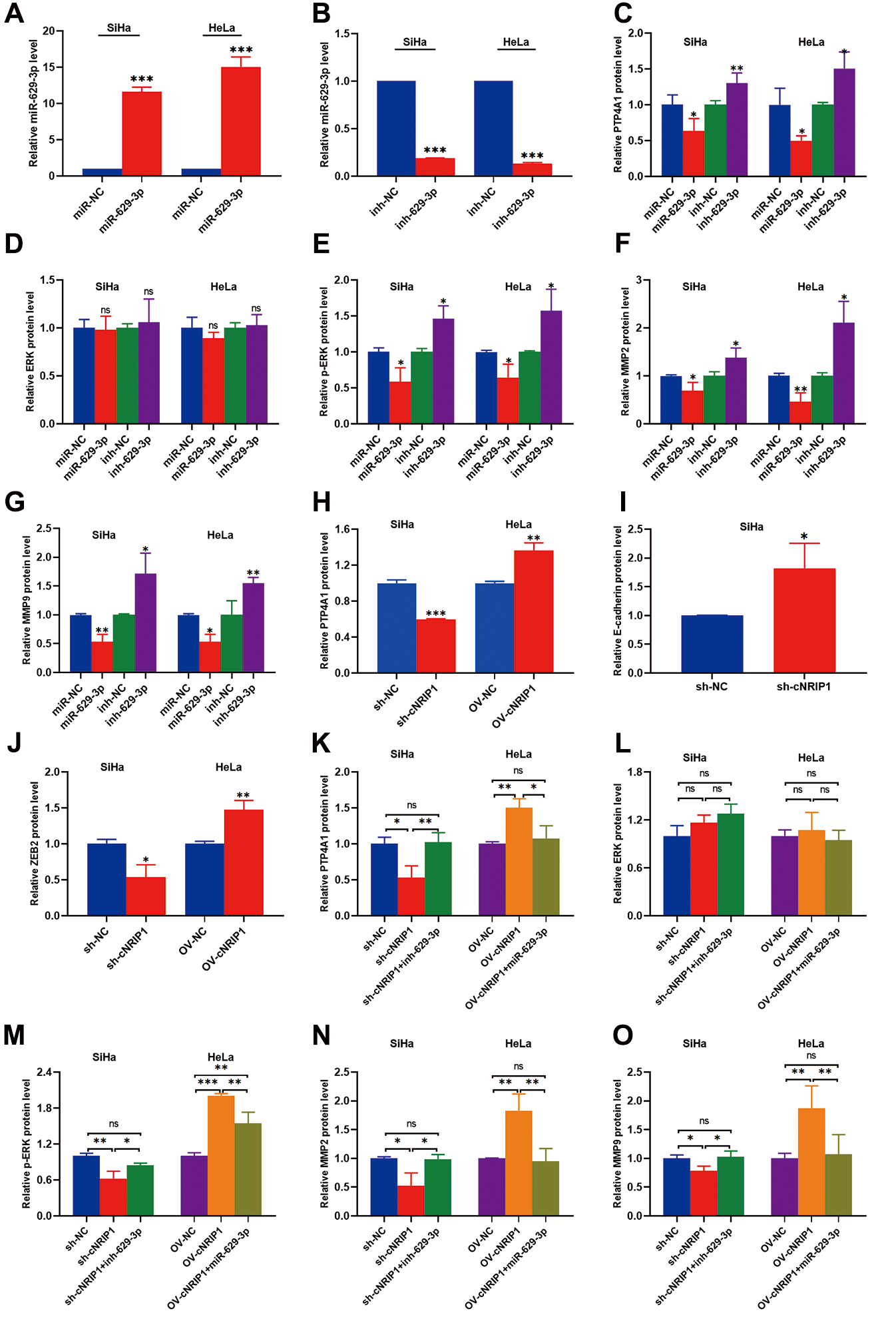

Supplement: Supplementary file 4 — Figure S3 [file 41419_2020_2607_MOESM4_ESM.png]

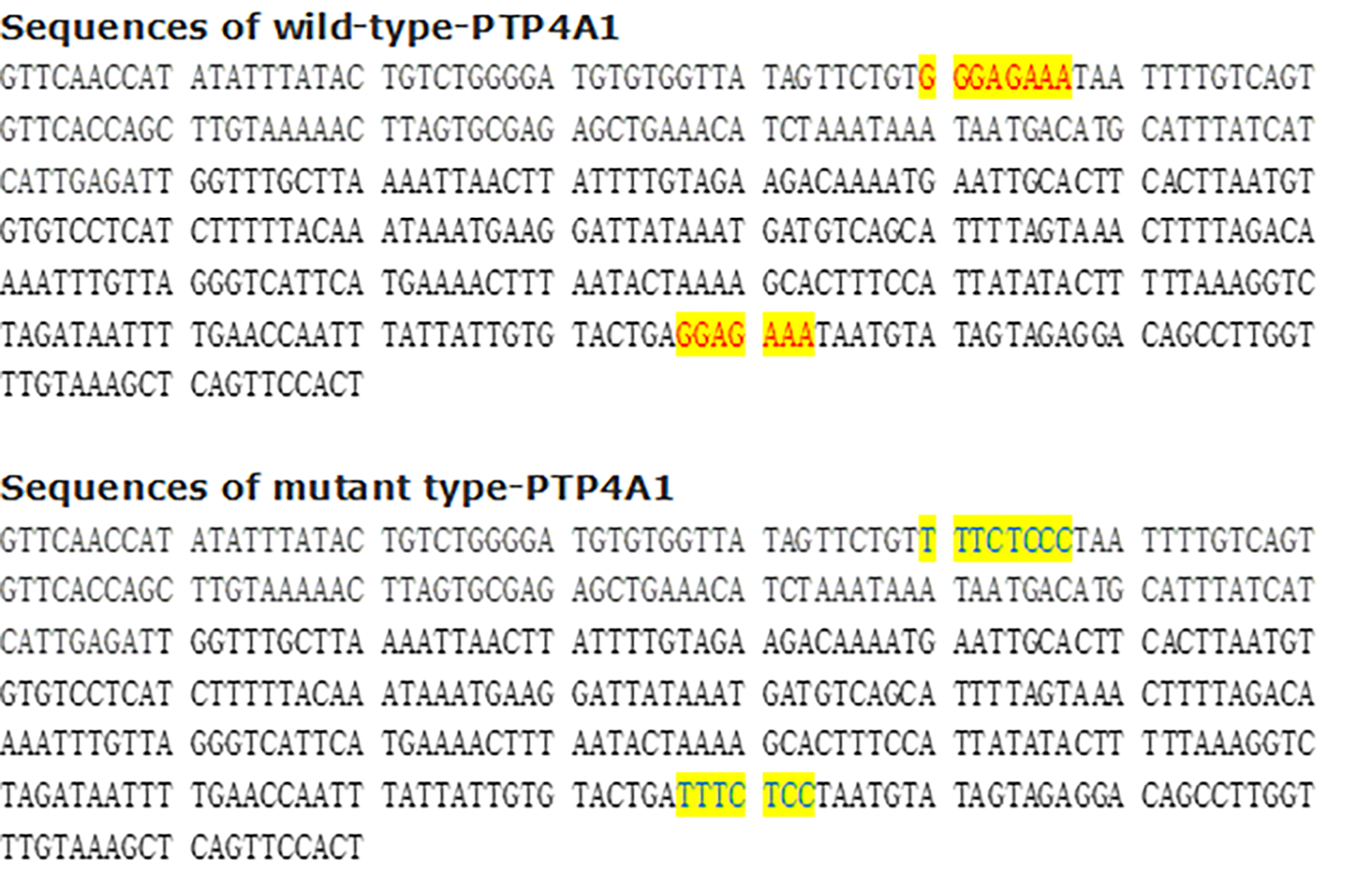

Supplement: Supplementary file 5 — Figure S4 [file 41419_2020_2607_MOESM5_ESM.png]
