# Supplementary material for: Circular RNA circNRIP1 promotes migration and invasion in cervical cancer by sponging miR-629-3p and regulating the PTP4A1/ERK1/2 pathway
Source: Cell Death Dis. 2020 May 26;11(5):399. doi: 10.1038/s41419-020-2607-9 (PMC7251091; doi:10.1038/s41419-020-2607-9)
Supplement: Supplementary file 6 — Table S1-S4 [file 41419_2020_2607_MOESM6_ESM.docx]

| **Table S1. Primer sequences for qRT-PCR** | | |
| --- | --- | --- |
| **Gene name** | **Forward primer** | **Reverse primer** |
| **Hsa_circ_0004771** | TTCTCAGAAAGCAGAGGCTCAG | GGCTGTGTTTCTCCCAAATGTT |
| **Hsa_circ_0003221** | CGGCGATCATACTGGGAGAT | AGTTGGGGTCAAGGTAAGCAG |
| **Hsa_circ_0002483** | ACCAGTTTACTGAAGATAAGCCA | GGTGTGTGATTCAAGTTGGGG |
| **Hsa_circ_0008460** | CGTTGGTGATTTGGTGTGGTC | GCCCAGATGCTTCCGTTCTT |
| **Hsa_circ_0006646** | AGGAGGTTCACTGGCTTCAC | ACAGATACCTAGCATCTAGCAA |
| **NRIP1** | GGATCAGGTACTGCCGTTGAC | CTGGACCATTACTTTGACAGGTG |
| **GAPDH** | ACAACTTTGGTATCGTGGAAGG | GCCATCACGCCACAGTTTC |
| **U6** | CGCTTCGGCAGCACATATAC | TTCACGAATTTGCGTGTCATC |
| **Hsa-miR-653** | GCGCGGTGTTGAAACAATCT | GTCGTATCCAGTGCAGGGTCCGAGGTATTCGCACTGGATACGACCAGTAG**(RT)** |
| **Hsa-miR-629-3p** | GCGGTTCTCCCAACGTAAG | GTCGTATCCAGTGCAGGGTCCGAGGTATTCGCACTGGATACGACGCTGGG**(RT)** |
| **Hsa-miR-149-5p** | CGTCTGGCTCCGTGTCTTC | GTCGTATCCAGTGCAGGGTCCGAGGTATTCGCACTGGATACGACGGGAGT**(RT)** |
| **Hsa-miR-330-5p** | GCGTCTCTGGGCCTGTGTC | GTCGTATCCAGTGCAGGGTCCGAGGTATTCGCACTGGATACGACGCCTAA**(RT)** |
| **Hsa-miR-339-5p** | CGTCCCTGTCCTCCAGGAG | GTCGTATCCAGTGCAGGGTCCGAGGTATTCGCACTGGATACGACCGTGAG**(RT)** |
| **Hsa-miR-595** | GCGGAAGTGTGCCGTGGT | GTCGTATCCAGTGCAGGGTCCGAGGTATTCGCACTGGATACGACAGACAC**(RT)** |

| **Table S2. sequences for sh-RNAs** | | | |
| --- | --- | --- | --- |
| **Name** | **sh-RNA sequences** | **Top strand** | **Bottom strand** |
| **shRNA-NC** | TTCTCCGAACGTGTCACGTAA | GATCCG**TTCTCCGAACGTGTCACGTAA**TTCAAGAGATTACGTGACACGTTCGGAGAATTTTTTC | AATTGAAAAAA**TTCTCCGAACGTGTCACGTAA**TCTCTTGAATTACGTGACACGTTCGGAGAACG |
| **shRNA1** | TTGGAGACAGA**CGGAAG**TGTTTGGA | GATCCG**TTGGAGACAGACGGAAGTGTTTGG**ATTCAAGAGATCCAAACACTTCCGTCTGTCTCCAATTTTTTG | AATTCAAAAAA**TTGGAGACAGACGGAAGTGTTTGGA**TCTCTTGAATCCAAACACTTCCGTCTGTCTCCAACG |
| **shRNA2** | GACAGA**CGGAAG**TGTTTGGATTGTG | GATCC**GACAGACGGAAGTGTTTGGATTGTG**TTCAAGAGACACAATCCAAACACTTCCGTCTGTCTTTTTTG | AATTCAAAAAA**GACAGACGGAAGTGTTTGGATTGTG**TCTCTTGAACACAATCCAAACACTTCCGTCTGTCG |
| **shRNA3** | GAGCTTGGAGACAGA**CGGAAG**TGTT | GATCC**GAGCTTGGAGACAGACGGAAGTGTT**TTCAAGAGAAACACTTCCGTCTGTCTCCAAGCTCTTTTTTG | AATTCAAAAAA**GAGCTTGGAGACAGACGGAAGTGTT**TCTCTTGAAAACACTTCCGTCTGTCTCCAAGCTCG |

| **Table S3. Sequences of oligonucleotides** | |
| --- | --- |
| **Name** | **Sequences** |
| **hsa-miR-629-3p mimics** | GUUCUCCCAACGUAAGCCCAGC |
|  | UGGGCUUACGUUGGGAGAACUU |
| **mimics negative control** | UUCUCCGAACGUGUCACGUTT |
|  | ACGUGACACGUUCGGAGAATT |
| **hsa-miR-629-3p inhibitors** | GUUCUCCCAACGUAAGCCCAGC |
|  |  |
| **Inhibitors negative control** | UUCUCCGAACGUGUCACGUTT |
|  |  |

| **Table S4. Five up-regulated circRNAs** | | | | | | |
| --- | --- | --- | --- | --- | --- | --- |
| **probeID** | **Fold Change** | **Log Fold change** | **Alias** | **chrom** | **GeneSymbol** | **Sequence** |
| **ASCRP3009450** | **8.4851267** | **3.0849362** | **hsa_circ_0004771** | **chr21** | **NRIP1** | **GGAGACAGACGGAAGTGTTTGGATTGTGAGCTATTTCAGAACTGTTCTCAGGACTCATTA** |
| ASCRP3011559 | 2.9892843 | 1.5798001 | hsa_circ_0002483 | chr8 | PTK2 | TCTATCAACAGAATATGACAGATACCTAGCATCTAGCAAAATAATGGCAGCTGCTTACCT |
| ASCRP3001950 | 2.6187434 | 1.3888747 | hsa_circ_0003221 | chr8 | PTK2 | AGATGCGGGGCAATGCACTAGAAAAGAAGTCTAACTATGAAGTATTAGAAATATGACAGA |
| ASCRP3008538 | 3.1971529 | 1.6767877 | hsa_circ_0008460 | chr4 | WHSC1 | GATCCACTCCTTCACAGCTATACCAAACTTAAAGTGTTCTAAGAACGGAAGCATCTGGGC |
| ASCRP3009197 | 2.9983025 | 1.5841459 | hsa_circ_0006646 | chr8 | PTK2 | GCTCACCCACCAGAGGAGTGGAAAATATGACAGATACCTAGCATCTAGCAAAATAATGGC |
